# Assessing and improving radiation safety in cardiac catheterization: a study from Cairo University Hospital

**DOI:** 10.1186/s43044-024-00449-7

**Published:** 2024-02-09

**Authors:** Muhammad Ghallab, Magdy Abdelhamid, Mahmoud Nassar, Karim S. Mostafa, Dina H. Salama, Wael Elnaggar, Shaban Alramlawy, Zakaria Alagha, Salma Abdelmoteleb, Assem Hashad

**Affiliations:** 1 Department of Cardiovascular Medicine, Cairo University, Cairo, Egypt; 2 Department of Internal Medicine, Icahn School of Medicine at Mount Sinai, New York, USA; 3 Radiology and Medical Imaging Technology Department, Misr University for Science and Technology, Cairo, Egypt; 4 Department of Critical Care Medicine, Cairo University, Cairo, Egypt; 5 Marshal University, Huntington, WV USA; 6 Cairo University School of Medicine, Cairo, Egypt; 7 Department of Medicine, Division of Endocrinology, Diabetes and Metabolism, Jacobs School of Medicine and Biomedical Sciences, University of Buffalo, New York, USA

**Keywords:** Cardiac catheterization, Radiation safety, Radiation protection, Radiation safety compliance

## Abstract

**Background:**

Catheter laboratories are high-radiation exposure environments, especially during X-ray procedures like percutaneous transluminal coronary angioplasty and electrophysiological studies. Radiation exposure poses risks of stochastic (e.g., cancer) and deterministic (e.g., skin changes) effects. This study assessed radiation safety and health practices in a cardiac catheterization unit to optimize radiation safety. A cross-sectional study in Cairo University Hospital (March–September 2019) evaluated 700 patients and healthcare workers. Real-time radiation measurements, educational lectures, and radiation protection measures were implemented in three phases. Data on radiation exposure, procedures, and compliance were collected and analyzed.

**Results:**

The total procedure time and fluoroscopy time per cardiologist did not significantly differ between phases, but there was a statistically significant reduction in the mean total cumulative radiation doses between Phase I and Phase III for cardiologists (*P* = 0.013). Among nurses and technicians, there was no significant difference in radiation doses between the two phases. Significant correlations were found between operators' radiation doses, procedure time, and fluoroscopy time. Patients' radiation doses decreased significantly from Phase I to Phase III, with correlations between dose, procedure time, and gender. Compliance with radiation protection measures was suboptimal.

**Conclusions:**

Compliance with radiation safety standards in the cardiac catheterization unit at the Cairo University Hospital needs improvement. The study highlights the importance of adhering to radiation safety principles and optimizing protective measures to reduce radiation exposure for both patients and healthcare personnel. Despite low compliance, significant reductions in radiation doses were achieved with increased awareness and adherence to specific protection measures. Future efforts should focus on enhancing radiation safety protocols and organ-specific radiation impact assessments.

## Background

Catheter laboratories, where procedures like coronary angioplasty and electrophysiological studies are performed using X-ray imaging, expose patients and medical staff to high levels of ionizing radiation [1, 2]. This radiation carries risks, categorized into deterministic effects, like skin issues and cataracts, and stochastic effects, increasing the likelihood of cancer with higher doses [2]. Workplace radiation exposure is limited to 20–50 millisieverts (mSv) per year [3].

Research shows that even a dose of 10 mSv can lead to five additional cancer deaths in 10,000 individuals [4]. Protective measures in catheter laboratories include leaded aprons, overhead shields, specialized glasses, and thyroid collars. Minimizing exposure involves reducing radiation duration and maintaining safe distances [5]. The ALARA principle guides keeping doses "as low as reasonably achievable" while maximizing benefits [6].

Catheter laboratories use fluoroscopy and cine techniques with varying exposure levels. Fluoroscopy guides catheters, while cine records procedures, resulting in higher exposure but for shorter durations. Some laboratories limit fluoroscopy frames to reduce exposure. On average, a coronary angiography exposes to about 120 chest X-rays, and percutaneous transluminal coronary angioplasty (PTCA) exposes to around 200 chest X-rays [1].

Recent reports show a 700% surge in radiation doses from clinical imaging in the USA between 1980 and 2006, raising concerns about the collective radiation exposure dose for the population [7]. Cardiologists need to carefully justify procedures and optimize radiation doses during tests to address these issues.

This study aims to comprehensively assess radiation safety and health practices in the cardiac catheterization unit at Cairo University Hospital. Radiation-induced skin reactions, though usually mild, can occur, and the study recognizes the necessity of some interventions, even if they pose risks [8].

## Methods

In 2019, a cross-sectional observational study was conducted in the Cardiology department at Cairo University Hospital, spanning from March to September. This study focused on evaluating radiation doses during diagnostic or interventional cardiac catheterization procedures, involving patients and unit staff.

The protocol gained approval from the Institutional Review Boards of the "Research Ethics Committee of the Faculty of Medicine at Cairo University" on January 15, 2019. All participants, including operators, nurses, technicians, and patients, provided informed written consent to partake in the study.

The study encompassed 700 patients, nine cardiologists, six nurses, and three technicians directly exposed to radiation during diagnostic coronary angiographies and percutaneous coronary interventions (PCI), excluding those not frequently working in the cardiac catheterization unit.

Structured into three phases, the study began with Phase I [8], involving real-time radiation exposure measurements for all stakeholders in the Cardiac Catheterization Laboratory. The key metrics assessed in the study were the—total procedure time, fluoroscopy time, cumulative radiation doses, and compliance with radiation protection measures.

Phase II (4 weeks) introduced educational lectures on radioprotection principles and associated hazards to Cath lab operators, nurses, and technicians. Finally, Phase III (12 weeks) encouraged operators to use radiation protection shields, with subsequent measurements comparing radiation exposure levels to those recorded during Phase I. This meticulous approach aimed to comprehensively understand the impact and effectiveness of radiation protection measures implemented in the cardiac catheterization unit at Cairo University Hospital during the specified period.

### Intervention

During Phase II, educational lectures on general radioprotection principles in the Cath lab and radiation exposure hazards were delivered to all operators, nurses, and technicians. The content of these lectures was sourced from "The American Interventional Cardiology Curriculum on X-ray Imaging and Radiation Safety" and the official website of the International Atomic Energy Agency (IAEA), focusing on pertinent Cath lab radioprotection topics displayed within the Cardiac catheterization unit.

### Data collection and measurements

*For the nine operators*, data collection encompassed the following parameters: total radiation exposure dose, number of procedures (diagnostic coronary angiographies/PCI), procedure type (Elective/Emergency), total fluoroscopy time, and total procedure time.

*For the patients*, the following data points were collected: radiation dose (measured by total radiation dose or Air kerma in milligrays (mGy) and dose area product (DAP) in microgray square meters), number of fluoroscopy machine exposures per procedure, and procedure duration in minutes.

Personal dosimeters were employed to measure the radiation dose for the nine cardiologists, nurses, and technicians. These dosimeters were worn above the lead apron (above the left nipple) and provided by the Cesium department at Cairo University. The minimum detectable dose was 50 μSV, and the dosimeters measured beta, gamma, and X-ray radiation.

A “RADALERT100 Nuclear Radiation Monitor” survey meter was employed to measure radiation doses once for each procedure in the Cath lab. Additionally, radiation dose (µsv/h) measurements were taken at various locations in the catheterization laboratory and control room to assess safety.

### Compliance assessment of staff unit

A self-administered questionnaire was developed based on a review of existing literature to evaluate the adherence of unit personnel to radiation protection measures. The questionnaire's validity was reviewed by a radiodiagnosis expert. Responses were rated on a four-point Likert scale, with four denoting "always," three for "often," two for "sometimes," and one for "never." Higher scores indicated better adherence, with poor adherence defined as scores of 60% or lower, moderate adherence as scores ranging from 60 to 75%, and good adherence as scores exceeding 75%.

### Statistical methods and analysis

Data analysis was performed using IBM SPSS (Statistical Program for Social Science, version 21). Descriptive statistics included mean, standard deviation, median, and interquartile range (IQR) for quantitative variables, while qualitative variables were expressed as numbers and percentages. For comparisons between groups, the Chi-square test was employed for qualitative variables, and the Mann–Whitney test replaced the unpaired t-test for non-parametric data (SD > 30%). For non-parametric data (SD > 30% mean), the Wilcoxon test replaced the paired *t* test. The Pearson Correlation coefficient test was utilized to assess linear associations between variables not following a normal distribution, with statistical significance defined as *p* < 0.05 as appropriate.

## Results

### The Cath lab staff radiation data

Table 1 presents various demographic and clinical characteristics. As depicted in Table 2, a total of 572 procedures were performed by nine cardiologists. Phase I involved 246 participants (mean: 31.67 ± 14.7), while Phase III included 326 participants (mean: 31.89 ± 18), with no statistically significant difference between the two groups (*P* = 0.906).

**Table 1:** Demographic and clinical characteristics of the Cath lab staff

No	Categories	Numbers	Age (years)	Experience (years)
1	Cardiologists	9	37.78 ± 2.99	5.67 ± 2.55
2	Nurses	6	40.8 ± 8.13	17 ± 8.9
3	Technicians	3	36.6 ± 8.1	14.3 ± 9.3

**Table 2:** Descriptive data on the procedures performed by the 9 cardiologists in Phases 1 and 2

No		Phase I Mean ± SD	Phase III Mean ± SD	*P* value
1	Total number of procedures	246	326	–
2	Number of procedures per cardiologist	31.67 ± 14.7	31.89 ± 18.07	0.906
3	Total time of procedures minutes per cardiologist	1729.07 ± 822.09	1577.44 ± 983.07	0.374
4	Total fluro time minutes per cardiologist	691.2 ± 397.82	594.56 ± 425.5	0.066
5	Total number of emergency procedures per cardiologist	123	102	–
6	Number of emergency procedures per cardiologist	13.67 ± 6.34	11.33 ± 4.85	0.326
7	Total cumulative dose mSv per cardiologist	4.29 ± 1.26	3.48 ± 1.26	0.013 S
8	Total cumulative dose mSv per nurse	2.7 ± 1.7	2.4 ± 1.5	0.058
9	Total cumulative dose mSv per technician	2.9 ± 1.9	4 ± 2.8	0.109
10	Total number of diagnostic angiographies	113	123	
11	Diagnostic angiographies per cardiologist	12.56 ± 5.73	14.78 ± 9.95	0.553
12	Total number of PCI	172	154	
13	Number PCI per cardiologist	19.11 ± 10.46	17.11 ± 8.95	0.191

In Phase I, there were 113 diagnostic angiographies (mean = 12.56 ± 5.73), and Phase III had 123 (mean = 14.78 ± 9.95), with no statistically significant difference (*P* = 0.553). For PCIs, Phase I involved 172 cases (mean 19.1 ± 10.46), and Phase III had 154 cases (mean 17.1 ± 8.95), also without statistically significant differences (*P* = 0.191). Emergency procedures (Primary PCI) constituted 40% of all procedures, with Phase I comprising 123 cases (mean 13.67 ± 6.34) and Phase III 102 cases (mean 11.33 ± 4.85), again with no significant difference (*P* = 0.326).

Results related to the cardiologists:

The mean total procedure time per cardiologist was 1729 ± 822.09 min in Phase I and 1577.44 ± 983.07 min in Phase III, showing no statistical difference (*P* = 0.374). Similarly, the mean total fluoroscopy time per cardiologist was 691.2 ± 397 min in Phase I and 554.5 ± 425.5 min in Phase III, with no statistically significant difference (*P* = 0.066).

The average total cumulative radiation dose (mSv) for the nine cardiologists in Phase I was 4.29 ± 1.26 and 3.48 ± 1.26 in Phase III, indicating a statistically significant difference (*P* = 0.013).

Results related to the nurses:

In Phase I, nurses received radiation doses ranging from 0.5 to 5.5 mSv (mean = 2.7 ± 1.7), and in Phase III, doses ranged from 0.6 to 4.8 mSv (mean = 2.4 ± 1.5). Although the difference in the total cumulative radiation dose exposure among the six nurses was not statistically significant (*P* = 0.058), it is noteworthy.

Results related to the technicians:

In Phase I, technicians received radiation doses ranging from 0.7 to 4 mSv (mean = 2.9 ± 1.9), while in Phase III, doses ranged from 0.8 to 5.8 mSv (mean = 4 ± 2.8). The mean total cumulative radiation doses for the three technicians did not significantly differ between the two phases (*P* value = 0.109).

**Fig. 1:**
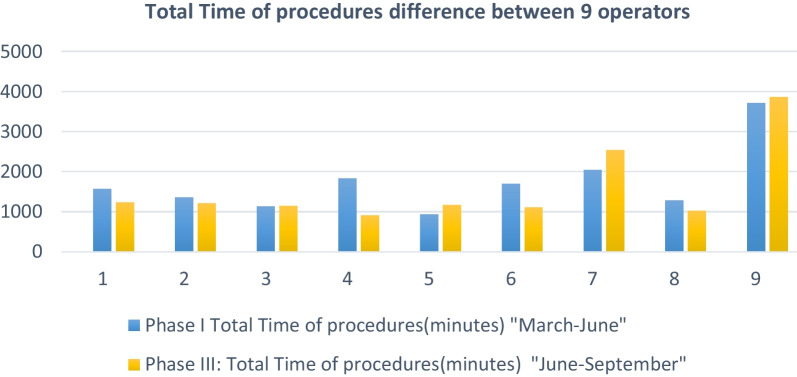
Total fluoroscopy time difference between 9 operators

**Fig. 2:**
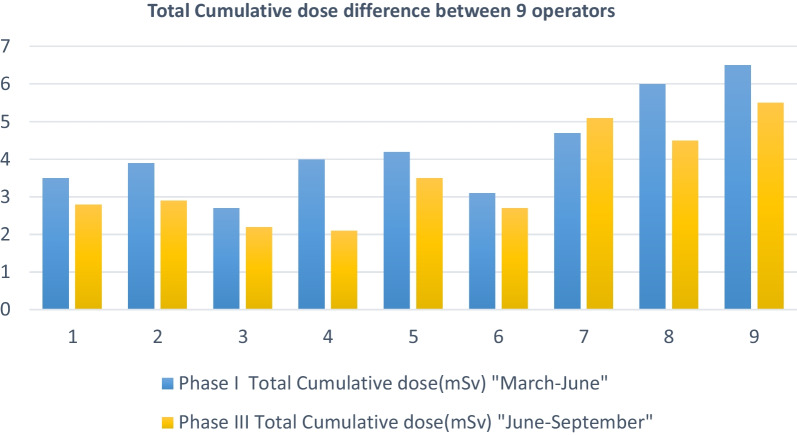
Total cumulative dose difference between 9 operators

Comparison among the nine operators revealed that in Phase I, the total number of procedures ranged from 18 to 65, and in Phase III, it ranged from 17 to 75. Notably, operator number 9 performed 139 procedures. Total procedural time for cardiologists in Phase I ranged from 900 to 3800 min and from 850 to 3900 min in Phase III. Operators number 7 and 9 had the longest procedural times (see Fig. 1). The total cumulative radiation doses in Phase I ranged from 2.7 to 6.5 mSv among the nine cardiologists, with a mean of 19.1 ± 10.46. In contrast, in Phase III, doses ranged from 2.1 to 5.5 mSv, with a mean of 3.48 ± 1.26 (see Fig. 2).

### Correlation between total cumulative radiation doses received by operators and different factors

Table 3 outlines the correlation between cumulative total radiation doses received by different cardiologists and various factors. In Phase I, no statistically significant correlation was observed between the total cumulative radiation doses received by the operators and any of the examined factors, including operators' age, work experience duration, total procedure count, total fluoroscopy time, number of diagnostic angiography procedures, or number of PCI procedures. However, in Phase III, two factors showed a statistically significant correlation with the total cumulative radiation dose: total procedural time (*P* value = 0.05) and total fluoroscopy time (*P* value = 0.03). Apart from these, no statistically significant correlations were found between the total cumulative radiation doses and other factors, including work experience duration, number of diagnostic angiography procedures, or PCI procedures.

**Table 3:** Correlation between total cumulative radiation doses received by the operators and different factors in Phase I and III

	Total cumulative radiation dose (mSv)			
	Phase I		Phase III	
	*r*	*P* value	*r*	*P* value
Duration of work experience (years)	− 0.301	0.43	− 0.08	0.838
Total number of procedures	0.218	0.574	0.445	0.23
Total time of procedures (min)	0.367	0.332	.667	0.05
Total fluoroscopy time (min)	0.417	0.265	.717	0.03
Total number of emergency procedures	0.218	0.572	0.335	0.379
Number of diagnostic angiography procedures	0.151	0.698	0.533	0.139
Number of PCI procedures	0.192	0.62	0.504	0.166

### Adherence to radiation protection practices by the operators

Tables 4 and 5 summarize operators' adherence to radiation protection measures and standards. During fluoroscopy, adherence to protective measures, such as wearing a thermoluminescent detector (TLD), a lead apron, and a thyroid collar, was suboptimal. Specifically, 55.5% of cardiologists neglected or rarely wore thyroid collars, and 77.7% reported sporadic TLD usage. Regarding patient protection practices, low adherence rates were observed in the use of lead (fixed table-suspended drapes or lead curtains between the X-ray tube and the operator) and glass shields (Ceiling-suspended movable screen to shield the operator from scatter radiation coming from the patient), with only 11.1% of participants frequently using them. Concerning practices related to environmental radiation protection, although most operators closed the doors during examinations (77.7%), they often did not keep them closed throughout the procedure.

**Table 4:** Adherence to radiation protection practices by cardiologists

Practices of participants regarding radiation protection	Response of the studied cardiologists (*n* = 9)							
	Never		Sometimes		Most of time		Always	
	No	%	No	%	No	%	No	%
Personal protection								
Wearing TLD during the work	0	0	7	77.7	2	22.2	0	0
Wearing lead apron during fluoroscopy	0	0	0	0	0	0	9	100
Wearing thyroid collar at the Cath lab	5	55.5	2	22.2	2	22.2	0	0
Patient protection								
Using lead shield	0	0	8	88.8	1	11.1	0	0
Using glass shield	6	66.7	2	22.2	1	11.1	0	0
Using minimum exposure time	3	33.3	2	22.2	4	44.4	0	0
Protection of the environment								
Closing the door room	0	0	2	22.2	7	77.7	0	0

**Table 5:** Scoring of the adherence to radiation protection practices among cardiologists

Score (%) of adherence toward personal protection	Studied cardiologists (*N* = 9)	
Practices related to operators personal protection (%)		
Min–max, mean ± SD	58–83.3	65.5 ± 9.87
Poor adherence (*N*%)	5	55.5
Moderate adherence (*N*%)	3	33.3
Good adherence (*N*%)	1	11.1
Practices related to patient protection (%)		
Min–max, mean ± SD	33.3–58	44.3 ± 8.3
Poor adherence (*N*%)	9	100
Moderate adherence (*N*%)	0	0
Good adherence (*N*%)	0	0
Practices related to environmental protection (%)		
Min–max, mean ± SD	50–75	66.6 ± 12.5
Poor adherence (*N*%)	3	33.3
Moderate adherence (*N*%)	6	66.7
Good adherence (*N*%)	0	0
Total score (%)		
Min–max, mean ± SD	46.4–67.8	56.7 ± 7
Poor adherence (*N*%)	4	44.44
Moderate adherence (*N*%)	5	55.55
Good adherence (*N*%)	0	0

Results related to the patients:

Regarding patients’ data, as shown in Table 6, 700 patients underwent coronary angiographies and PCI procedures during the study, with 355 patients in Phase I and 345 patients in Phase III. No statistically significant differences were observed between Phase I and Phase III in terms of gender (*P* = 0.714) or age (Phase I: 55.4 ± 11.2 years, Phase III: 56.9 ± 9.9 years).

**Table 6:** Baseline demographic characteristics of the patients and type of the procedures done in Phases I and III

	Phase I	Phase III	*P* value
Sex	*n* (%)	*n* (%)	
Male	227 (63.9)	216 (62.6)	0.714
Female	128 (36.1)	129 (37.4)	
Type of procedure	*n* (%)	*n* (%)	
Coronary angiography	151 (42.5)	162 (47)	0.239
PCI	204 (57.5)	183 (53)	
Procedure urgency	*n* (%)	*n* (%)	
Elective	203 (57.2)	215 (62.3)	0.166
Emergency	152 (42.8)	130 (37.7)	

**Fig. 3:**
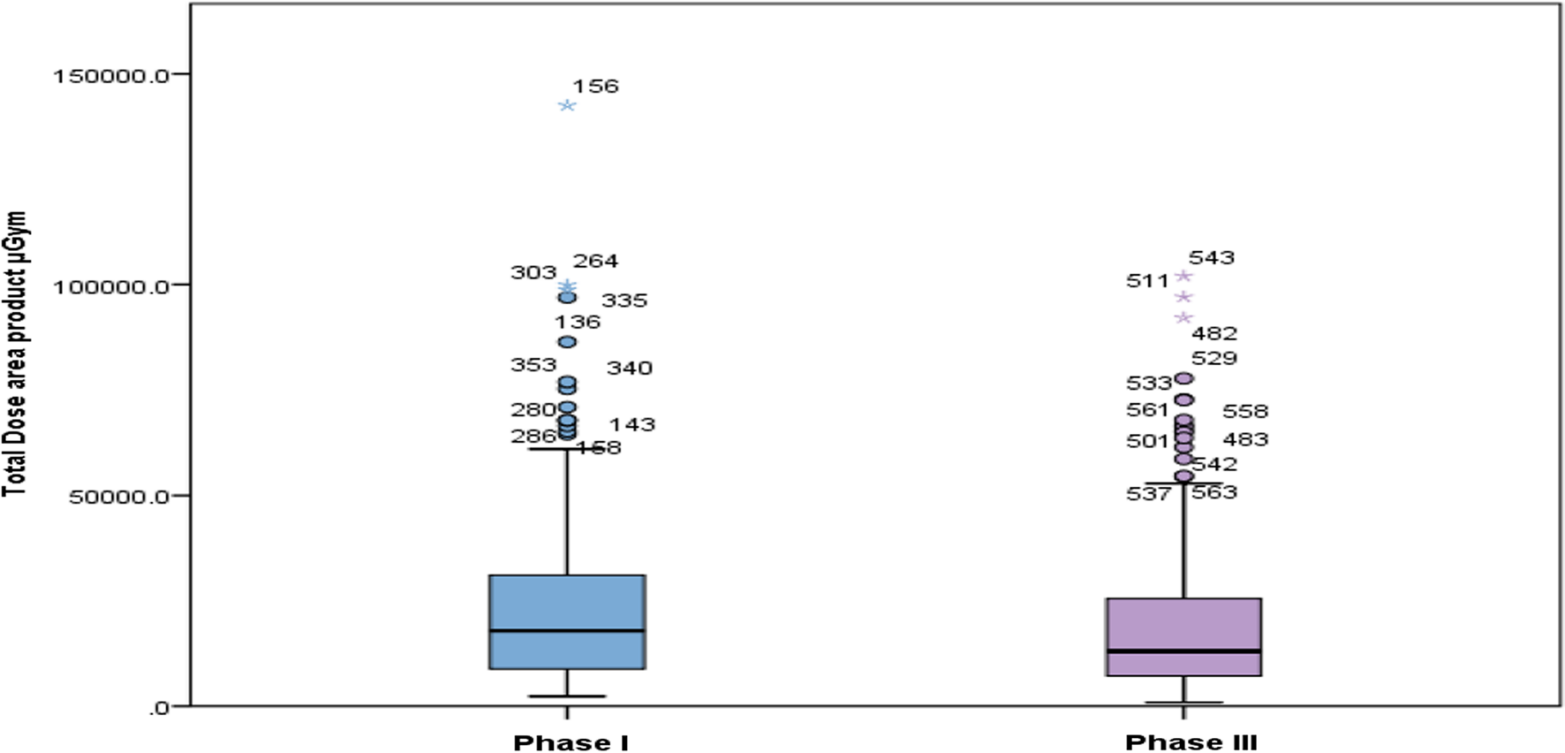
Difference between two phases regarding total dose area product

**Fig. 4:**
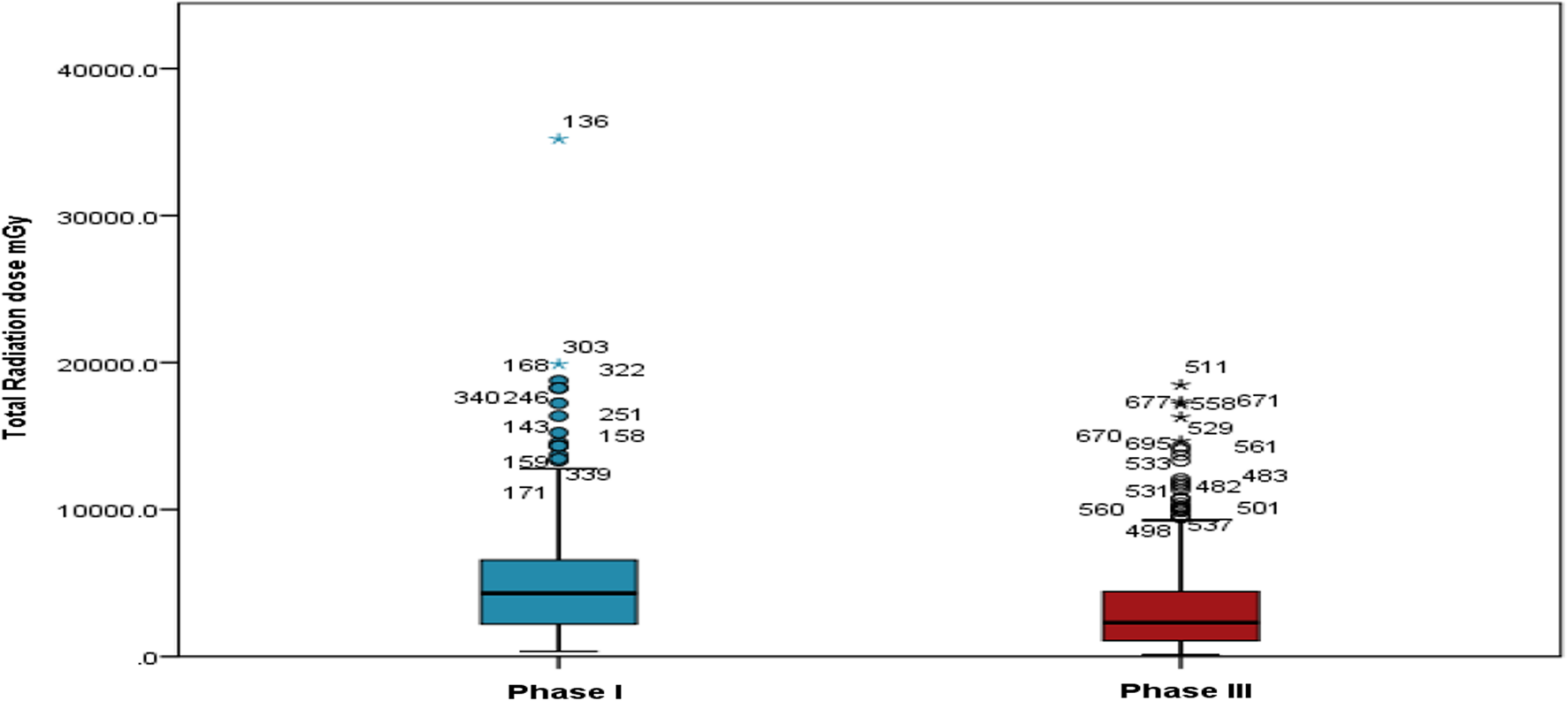
Difference between two phases regarding total radiation dose

**Fig. 5:**
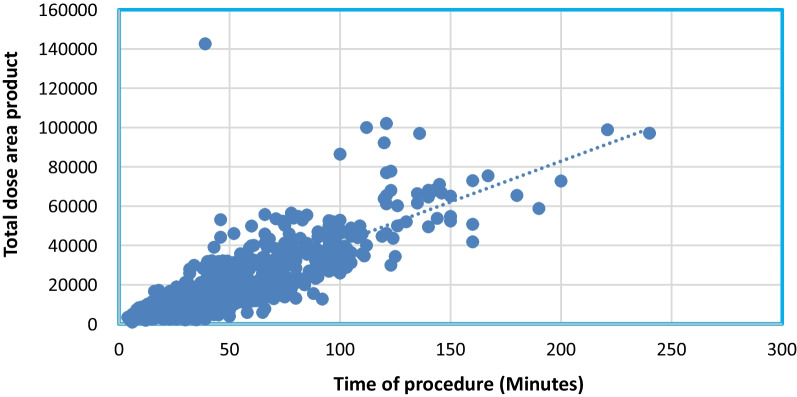
Correlation between the time of the procedure with total DAP in phase I

**Fig. 6:**
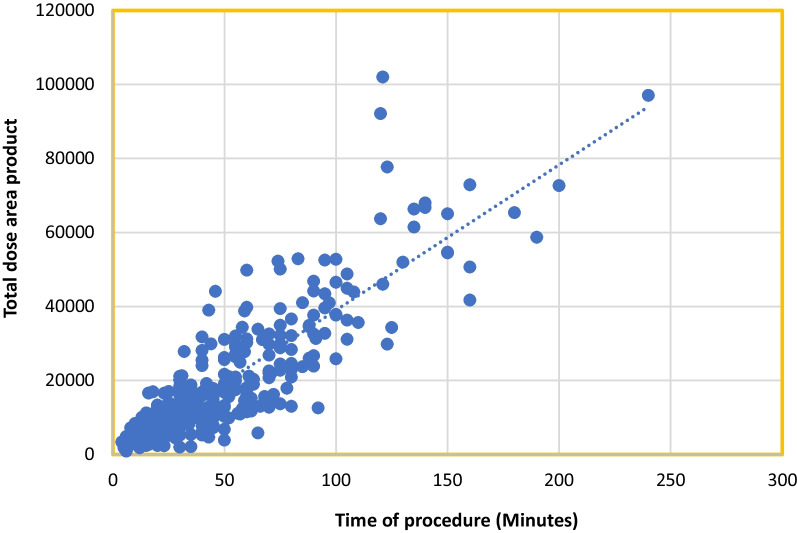
Correlation between the time of the procedure with total DAP in phase III

Highly significant differences were noted between the two phases in mean total dose area product (*P* < 0.001) “Fig. 3” and mean total radiation dose (Air kerma) (*P* < 0.001) “Fig. 4”. Furthermore, a strong correlation was observed between the total DAP and total procedure time in both phases (*P* < 0.001), as depicted in Figs. 5 and 6. A statistically significant correlation was also noted between the total radiation dose received by the patients and the total procedure time in both phases (*P* < 0.001). Additionally, there was a significant correlation between total DAP and total radiation dose in relation to fluoroscopy time in Phase I and Phase III (*P* < 0.001). Moreover, a statistically significant correlation between total DAP, total radiation dose received by the patients, and gender was identified, especially in males (*P* < 0.007, 0.019) in both Phase I and Phase III. These findings shed light on various aspects of radiation exposure, operator practices, and patient outcomes in the Cardiac Catheterization Laboratory.

## Discussion

We conducted a comprehensive study to assess radiation exposure in the Cardiac Catheterization Laboratory (Cath lab) for operators, nurses, technicians, and patients. This study represents the first of its kind in the Africa-Middle East region, shedding light on the critical role of radiation protection in reducing radiation exposure for patients and healthcare personnel in the Cardiac Catheterization Laboratory. Additionally, it evaluates the extent to which operators, nurses, and technicians adhere to radioprotective measures.

Understanding the importance of minimizing patient radiation exposure, which inherently reduces operator risk [9], is pivotal. It is essential to recognize the challenges associated with implementing radiation protection measures, given the invisible nature of radiation and its predominantly long-term effects [10]. These challenges became evident during the study, underscoring the significance of Phase II, aimed at heightening awareness among staff.

The Society for Cardiovascular Angiography and Interventions has consistently emphasized the importance of physicians possessing knowledge in radiation physics and X-ray machine operation, considering it their responsibility to comprehend this knowledge base. Simultaneously, hospitals are mandated to monitor and ensure worker safety, particularly regarding optimal occupational exposure to radiation [1]. Our initiative was prompted by the need to enhance radiation awareness and documentation.

An evaluation of radiation doses received by healthcare personnel revealed that the doses often exceeded annual radiation exposure limits. This observation may be attributed to our prevailing "Angiographic culture," which prioritizes the optimization of angiographic procedures while potentially overlooking radiation hazards for operators, patients, and the environment [11].

Numerous studies have highlighted the importance of incorporating shielding systems to primarily minimize the radiation dose received by operators [12, 13]. In a pre-clinical investigation conducted by Dixon et al. [12], a recently developed radiation shielding system demonstrated equivalent effectiveness to traditional lead aprons, leading to a notable decrease in scatter radiation doses.

In a recent publication, the cardiac catheterization team at the Cleveland Clinic shared their experience in radiation dose reduction. They implemented advanced protocols, including reducing the fluoroscopic frame rate to 7.5 frames per second, installing a low dose mode for acquisition on the foot switch, and introducing a pulse detector dose reduction. This protocol yielded a significant reduction in radiation exposure to patients, along with decreased air kerma and kerma area products [14].

Balancing the use of low radiation doses with the need for high-quality imaging for accurate diagnosis is crucial, aligning with the ALARA (As Low As Reasonably Achievable) and AHARA (As High As Reasonably Achievable) principles [15]. This implies that the goal is not to eliminate radiation exposure but to make it appropriate. Despite the absence of a statistically significant reduction in fluoroscopy time between Phases I and III, data from operator radiation exposure in these phases indicated a statistically significant reduction in the cumulative radiation dose in Phase III. This reduction could be attributed to the increased awareness fostered during Phase II, leading to greater utilization of radiation protection equipment, especially lead aprons and table protection shields.

Our findings revealed that the radiation dose exposure for nurses and technicians remained relatively unchanged between the two phases. Notably, nurses' exposure to radiation may surpass recommended limits and even exceed that of the operators [16]. This discrepancy may stem from a lack of awareness regarding factors influencing radiation dose, including proximity to the radiation source, tube geometry, procedure type, and time spent in the Cath lab [16]. Consequently, greater efforts are warranted to enhance awareness of radiation protection optimization among Cath lab personnel [17, 18].

Phase III unveiled a statistically significant correlation between the total radiation dose received by operators and both the total procedure time and fluoroscopy time. This correlation clarifies the elevated radiation dose received by operator number 9, who had the lengthiest procedural time, driven by a substantial caseload of complex coronary cases, including chronic total occlusion cases. This underscores the importance of implementing real-time radiation monitoring within the Cath lab, providing instantaneous auditory feedback and real-time reporting of operator radiation dose [10]. This technology's significance is supported by the results of the RadiCure randomized control trial, conducted by Christopoulos G. et al., which demonstrated a substantial reduction in operator radiation exposure in cardiac catheterization laboratories through the use of real-time radiation monitoring devices [10].

Our data further disclosed a noteworthy reduction in total radiation doses received by patients in Phase III, exhibiting a significant correlation with fluoroscopy time across both phases. This underscores the benefits of optimizing radiation protection practices for both healthcare personnel and patients. Patients are particularly susceptible to direct deterministic effects of radiation, rather than the long-term stochastic effects, with potential adverse effects on hair and skin, ranging from mild to severe [19].

The findings of our study revealed a significant challenge in terms of compliance with radiation protection measures among operators, nurses, and technicians in the Cardiac Catheterization Laboratory (Cath lab) at Cairo University Hospital. This challenge arose not only from a lack of awareness regarding radiation hazards but also from inadequate adherence to protective measures. Despite the suboptimal compliance observed, there was a notable reduction in radiation doses among operators when some attention was given to radiation protection measures. This juxtaposition suggests that while compliance may be an issue, the implementation of protective measures, even to a limited extent, can still yield positive outcomes in terms of reducing radiation exposure.

Our study's compliance results diverged from those reported by Abuzaid et al. in July 2017 at the University of Sharjah [20]. In our study, overall adherence scores ranged from 46.4 to 67.8%, with a mean score of 56.7 ± 7. In contrast, Abuzaid et al. [20] reported adherence scores ranging from 13.3 to 100.0%, with a mean score of 75.2% ± 18.5 in their study. These differences may be attributed to variations in the awareness programs, institutional policies, or cultural factors influencing compliance across different settings. The observed suboptimal compliance underscores the need for intensified efforts in educating and motivating healthcare personnel within the Cath lab about the importance of adhering to radiation protection measures. This includes raising awareness about the potential risks associated with radiation exposure, emphasizing the long-term benefits of compliance, and providing continuous training on the proper utilization of protective equipment.

Addressing the reasons behind the observed non-compliance is crucial for developing effective strategies. Possible contributing factors may include a lack of understanding about the potential harm caused by radiation, time constraints during procedures, or a perception that the protective measures may impede the efficiency of the cardiac catheterization processes. Interventions to enhance compliance could involve regular and targeted training sessions, incorporating real-life case scenarios to illustrate the impact of non-compliance on both healthcare personnel and patients. Additionally, fostering a culture of open communication within the Cath lab, where concerns and challenges related to radiation protection are openly discussed, can contribute to a collective commitment to compliance.

It is worth noting that achieving optimal compliance is an ongoing process that requires continuous monitoring, feedback, and reinforcement. Implementing a feedback loop that provides timely information on individual and collective compliance levels, coupled with recognition for adherence to protocols, can contribute to a positive shift in attitudes and behaviors regarding radiation protection within the Cardiac Catheterization Laboratory.

### Clinical implications and learning points

This study constituted a foundational assessment of compliance with radiation safety standards; however, it transcended mere evaluation and spurred a commitment to enhance practices within our department. Several measures were undertaken to enhance our radiation safety protocols, including:

1. *Implementation of Personal Thermoluminescent Dosimeters (TLDs)* The successful adoption of personal TLDs by most operators was a pivotal step in enhancing radiation monitoring and ensuring the safety of our team.
2. *Comprehensive Training* Rigorous training programs were conducted for operators, nurses, and technicians, focusing on identified areas of improvement. This initiative provided a comprehensive understanding of our department's radiation safety practices, enabling us to align more closely with international standards.
3. *Knowledge Sharing* Collaboration with other departments exposed to radiation was established to disseminate the insights gained from our study. This knowledge-sharing endeavor aimed to enhance quality control and improve outcomes across multiple departments.

### Recommendations

Based on the findings from the study, several recommendations can be made to improve radiation safety for both medical staff and patients. These recommendations aim to address specific issues identified in the study:

A. Recommendations for Cardiologists:
  1. *Procedure Optimization* Balance procedure distribution among cardiologists and evaluate and minimize procedural times to reduce cumulative radiation exposure. Also, limit the use of the acquisition (cine) to minimize the received radiation dose.
  2. *Continuous Training* Provide ongoing radiation safety training, and highlight the correlation between procedural times and radiation doses
  3. *Equipment Maintenance* Ensure regular calibration of fluoroscopy equipment
  4. *Monitoring and Feedback* Implement continuous monitoring of individual radiation doses and provide timely feedback to promote awareness and improvement.
  5. *Adherence to Protective Measures* Emphasize the importance of wearing protective lead aprons, and thyroid collars, using the protective glass shield all the time, and monitoring and enforcing protective measures during fluoroscopy.
  6. *Environmental Protection* Reinforce keeping doors closed throughout procedures.
B. Recommendations for Nurses and Technicians:
  1. *Training and Awareness* Provide radiation safety training and encourage awareness and adherence to safety protocols.
  2. *Regular Monitoring* Implement a system to track and analyze radiation doses and establish thresholds for cumulative doses, triggering reviews or additional training.
C. Recommendations for Patient Protection:
  1. *Educational Initiatives* Develop patient education programs on risks and benefits and encourage patient collaboration in safety.
  2. *Optimize Imaging Techniques* Explore and implement advanced imaging techniques.
  3. *Utilize Protective Shields* Promote routine use of shields to protect patients.
D. Overall Quality Improvement:
  1. Conduct periodic audits of safety practices and use the audit results for continuous improvement.
  2. Foster a collaborative culture among staff members and support ongoing research in radiation reduction technologies.

Implementing these recommendations will enhance radiation safety, minimize occupational exposure, and optimize patient care outcomes in the cardiac catheterization unit. Regular reviews and adjustments to protocols will contribute to a culture that prioritizes the well-being of both staff and patients.

### Limitations of the study

It is imperative to acknowledge certain limitations within our study including:

1. *Organ-Specific Radiation Impact* This study did not evaluate the impact of radiation exposure on specific organs, including the thyroid, eyes, bone marrow, and brain. These organs can be susceptible to serious complications, including premature cataracts. Consequently, the involvement of specialized ophthalmologists and organ-specific assessments is imperative in future studies.
2. The research is conducted at a single center, rather than multiple centers.
3. The nature of the study is cross sectional, which carries inherent limitations. Cross-sectional studies cannot establish a cause-and-effect relationship, are susceptible to selection and information bias, and are prone to confounding.
4. The study period was relatively short, leading to constraints on the selected sample size.

### Potential future research directions

To advance radiation safety in cardiac catheterization units, future research should focus on enhancing safety protocols and conducting detailed organ-specific radiation impact assessments. Key areas for exploration include the development of advanced protocols using artificial intelligence for real-time monitoring, tailoring radiation doses based on individual patient characteristics, and evaluating emerging technologies for dose reduction; including comprehensive data collection for operators that encompass factors such as the operator's experience, the number of procedures conducted by each operator, and the nature of the procedures including but not limited to chronic total occlusions (CTOs), bifurcations, structural interventions, and electrophysiological procedures, such as pacemaker installations which would augment the data collection process, ensuring a more detailed understanding of operator-related variables in the study. In-depth studies on the organ-specific impact of radiation, long-term follow-up of healthcare professionals, and research on patient-centric approaches are essential. Collaborative multi-center initiatives, integration of quality improvement strategies, and ethical considerations in research practices are crucial aspects to explore. By looking into these research areas, the scientific community may make a significant contribution to the continued development of radiation safety in cardiac catheterization facilities, creating a safer and more effective healthcare environment for all the staff and the patients involved.

## Conclusions

In comparison with international radiation protection standards, staff compliance within the cardiac catheterization unit at Cairo Hospital falls below the established benchmarks. To address this deficiency, heightened emphasis and dedicated efforts are warranted to optimize radiation safety measures.

Furthermore, the statistically significant correlations observed between fluoroscopy time, total procedure time, and the radiation dose received by both operators and patients underscore the critical importance of adhering to the As Low As Reasonably Achievable (ALARA) and As High As Reasonably Achievable (AHARA) principles in our radiation safety protocols.

Remarkably, despite the observed low compliance, the study revealed a significant reduction in individual radiation doses with modest attention to specific radiation protection measures. In light of these findings, it is evident that there is a pressing need for targeted interventions to improve compliance and reduce radiation exposure within the cardiac catheterization unit at Cairo Hospital. Here are specific recommendations and potential strategies based on the study's outcomes: Implement intensive education and training programs, continuous monitoring with feedback, foster a cultural shift toward radiation safety, explore advanced protocols, enhance real-time monitoring, and consider behavioral incentives to improve compliance and reduce radiation exposure in the cardiac catheterization unit at Cairo Hospital. By implementing these recommendations, Cairo Hospital can make significant strides toward improving compliance with radiation protection measures and ultimately reducing radiation exposure for both healthcare personnel and patients in the cardiac catheterization unit. It is crucial to approach these strategies comprehensively and collaboratively, involving all stakeholders in the process of enhancing radiation safety practices.

## Data Availability

All the study data and the used materials are available upon request. All the study data were de-identified, and access to study data was limited to only a few study team members.

## Abbreviations

Msv: Millisieverts
ICRP: The International Commission on Radiological Protection
CXR: Chest X-rays
IAEA: International Atomic Energy Agency
PCI: Percutaneous coronary interventions
mGy: Milligrey
DAP: Dose area product
TLD: Thermoluminescent detector
